# Estimation of Agricultural Water Consumption from Meteorological and Yield Data: A Case Study of Hebei, North China

**DOI:** 10.1371/journal.pone.0058685

**Published:** 2013-03-14

**Authors:** Zaijian Yuan, Yanjun Shen

**Affiliations:** 1 School of Economics & Management, Hebei University of Science and Technology, Shijiazhuang, China; 2 Center for Agricultural Resources Research, Institute of Genetics and Developmental Biology, Chinese Academy of Sciences, Shijiazhuang, China; National University of Singapore, Singapore

## Abstract

Over-exploitation of groundwater resources for irrigated grain production in Hebei province threatens national grain food security. The objective of this study was to quantify agricultural water consumption (AWC) and irrigation water consumption in this region. A methodology to estimate AWC was developed based on Penman-Monteith method using meteorological station data (1984–2008) and existing actual *ET* (2002–2008) data which estimated from MODIS satellite data through a remote sensing *ET* model. The validation of the model using the experimental plots (50 m^2^) data observed from the Luancheng Agro-ecosystem Experimental Station, Chinese Academy of Sciences, showed the average deviation of the model was −3.7% for non-rainfed plots. The total AWC and irrigation water (mainly groundwater) consumption for Hebei province from 1984–2008 were then estimated as 864 km^3^ and 139 km^3^, respectively. In addition, we found the AWC has significantly increased during the past 25 years except for a few counties located in mountainous regions. Estimations of net groundwater consumption for grain food production within the plain area of Hebei province in the past 25 years accounted for 113 km^3^ which could cause average groundwater decrease of 7.4 m over the plain. The integration of meteorological and satellite data allows us to extend estimation of actual *ET* beyond the record available from satellite data, and the approach could be applicable in other regions globally where similar data are available.

## Introduction

The most critical resource for agroecosystems in China is water [1]. The total annual water resources available in China are around 2,800 km^3^. With a population of 1.3×10^9^, the available water per capita is only 2,100 m^3^/y. Thus, China is a nation with high water scarcity compared to a global average of 6,466 m^3^/y [2]. Water resources in the northern parts of China account for less than 20% of the national total, whereas arable land accounts for 65% of the total [3], and the grain production in the North has exceeded to 50% since 2005. As 80% of China’s food is produced on irrigated farmland, irrigation water plays an important role in feeding the large population [4], [5]. The North China Plain (NCP) is one mostly important granary of China. It has 140,000 km^2^ of arable land and produces about 20% of the nation’s grain food.

The natural rainfall cannot meet crop water requirements in NCP, supplementary irrigation is therefore widely applied to increase yields and to secure the food supply for the nation [3]. However, excessive use of diverted river ﬂows and groundwater has caused severe environmental problems. For example, since 1972 the lower reaches of the Yellow River has frequently dried up during the dry seasons for several years. During the droughts of 1997 it didn’t reach the sea for even 228 days. However, it must be mentioned that since the beginning of the 2000s, after a river basin management plan approach was adopted in Yellow River Basin, no drying up has occurred so far [2].

On the other hand, in most places of the NCP, such as Hebei province, groundwater is the primary source of water for irrigation. Grain production in Hebei province totaled 2.9 × 10^10^ kg in 2008, accounting for 5.5% of the country’s total, while the production of wheat and corn shared for 10.9% and 8.7% of the national total, respectively. Due to continually over-pumping, groundwater resource has been greatly depleted and facing to great challenges in sustainability. The water table at the piedmont plain for example has declined rapidly from ∼10 m below ground surface in the 1970s to ∼30 m in 2001 [6], and to ∼40 m in 2010 [7].

It is extremely important for a sustainable agricultural water management to explicitly estimate the groundwater consumption for agriculture in recent decades in NCP. FAO Penman-Monteith equation combined with crop coefficient was widely used for estimation crop water requirement over the world. For the NCP region, Liu et al. [8] calculated the crop water requirement for winter wheat and summer maize in North China in the past 50 years and found a widely decreasing trend of −0.9 ∼ −19.2 mm per decade for wheat and −8.3 ∼ −24.3 mm per decade for maize, respectively. Li et al. [9] successfully estimated the water consumption and crop water productivity of winter wheat in NCP using remote sensing for a growing season in 2003–2004. Their calculation suggested the average water consumption (i.e. ET) by winter wheat in 83 counties was 424 mm, which was 118 mm higher than the precipitation. Yang et al. [10] estimated that the crop water requirement for five major crops (wheat, maize, cotton, fruit trees, vegetables) in NCP using crop models DSSAT and COTTON2K, and found wheat accounted for over 40% of total irrigation water requirement in the plain, while maize and cotton together accounted for 24% of the total irrigation water requirement. They also estimated that the annual averaged irrigation requirement for grain crops was 6.16 km^3^ during the period of 1986–2006. This estimation is of great importance to make regional water resources planning. Though the crop model with careful calibrations can provide relatively accurate estimation of crop water consumption, the difficulties in collecting huge amount of information on soil profiles and crops biology and phenology together with the complicated parameterization restrict the wide application of crop model to regional water resources management, especially for the regions with limited data. Moreover, even in some developed countries, actual evapotranspiration (*ET*) has been observed only in recent 1–2 decades, mostly at field scale. Simple methods to estimate agricultural water consumption (AWC) at larger spatial and temporal scales are urgently needed for water resources assessment and planning. In the present study, we attempt to propose a simple method to calculate long-term regional AWC by using limited meteorological and census data.

Therefore, the main objectives of the present study are to estimate 1) the AWC changes over past decades in Hebei province; and 2) irrigation water consumption for agriculture and related groundwater depletion. The results from this study will provide critical information for the future development of sustainable agricultural water resources management practices for local governments.

## Materials and Methods

**Figure 1:**
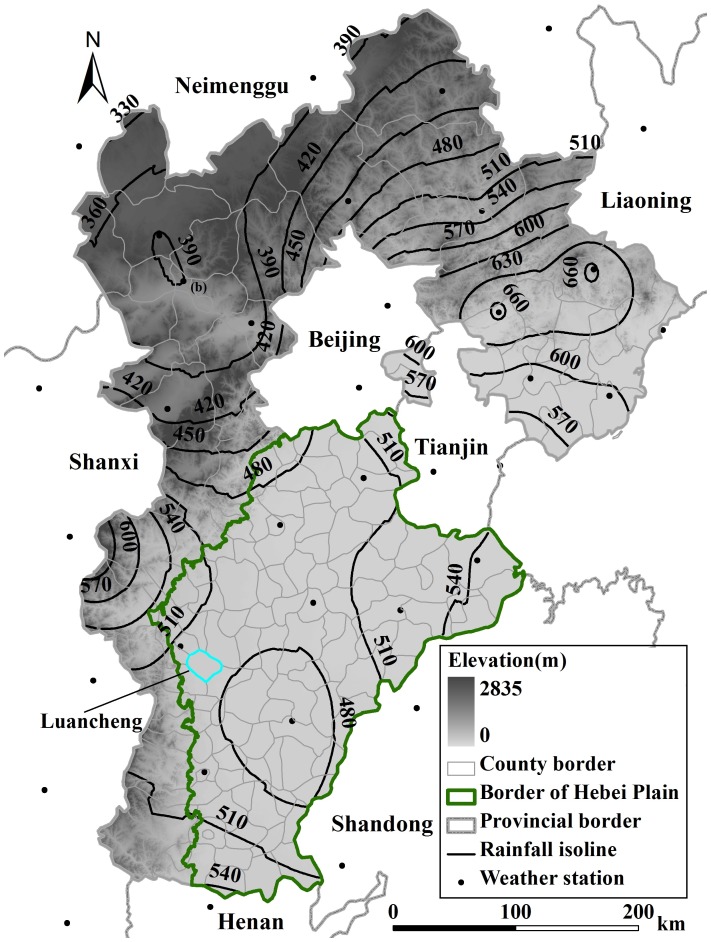
Geographical position of Hebei province. The contour lines and the points indicate average precipitation (1984–2008) and locations of weather stations used in this study, respectively.

Hebei province (36°05′N-42°40′N, 113°27′E-119°50′E, Figure 1) is 190,000 km^2^ in area with a population of 69 million (2009), and is divided into 11 prefectures (including 138 counties). The topography consists of mountains, hills, and plateaus in the north and west part, and a broad plain in the central and southeastern region. 34% of the provincial land area is cultivated with grain crops such as wheat, maize, rice, soybean, potato and millet, and among them the yield of winter wheat and summer maize account for 85% of the total grain yield (winter wheat is cultivated from early October to early June, summer maize grows from mid-June to late September). In plain area, most arable lands are irrigated except for the eastern part where the saline shallow groundwater restrains the irrigation but irrigation increased gradually in recent 3 decades due to technology evolution.

The study area is located in a temperate and continental monsoon climate zone with a mean annual precipitation of 500 mm (1984–2008), 70% of which occurs between June and September. Mean annual temperature is 10°C (1984–2008). Precipitation and temperature decrease from southeast to northwest.

### Data

The meteorological data for 1984–2008, including daily average temperature, relative humidity, precipitation, sunshine duration, atmospheric pressure, vapor pressure, wind speed, were obtained from 55 national weather stations (Figure 1). The economic statistics data for each county from 1984 to 2008, including grain yield, sowing area and effective irrigation area, were obtained from Hebei economic statistical yearbooks. The meteorological data were used to calculate reference evapotranspiration, and economic data were employed to estimate the actual evapotranspiration.

An independent remote sensing ET dataset was employed to analyze the relationship between grain yield and ET and to calibrate a key parameter, i.e. *K_f_*, of the model we proposed. The remote sensing *ET* data were produced based on moderate-resolution imaging spectroradiometer (MODIS) data by combining meteorological records and an scheme called ETWatch. There are 7 years (2002–2008) *ET* data available for Hebei province with a 1 km spatial resolution. Wu et al. [11] presented the details of the algorithm of ETWatch and its validation.

Validation data are collected from five years (2007–2011) field experiments on irrigation and water productivity at Luancheng Agro-ecosystem Experimental Station (35°53′N, 114°41′E), the Chinese Academy of Sciences, which is located at the piedmont, with an elevation of 50 m above sea level. The experiments have been conducted in 16 water balance plots with an area of 50 m^2^ each. Irrigation was applied as five treatments to control the soil moisture at different levels (see Sun et al. [12] for details). The data of annual irrigation amount, annual total yield of the double crops wheat and maize, actual *ET* calculated from soil water balance for each treatment were collected as well as the daily meteorological data and groundwater depth monitoring data. The meteorological data was used to calculate the reference *ET* at this station, other data were employed to validate and evaluate the model’s applicability.

### Reference Evapotranspiration

Reference evapotranspiration was estimated through FAO56-PM model [13],
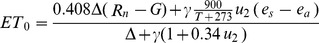
(1)where *ET_0_* is reference evapotranspiration (mm d^−1^) and annual *ET_0_* was accumulated from daily *ET_0_*; *R_n_* is the net radiation at the crop surface (MJ m^−2^d^−1^); *G* is the soil heat flux density (MJ m^−2^d^−1^); *T* is daily average temperature (°C); *u_2_* is the wind speed at 2 m height (m s^−1^); *e_s_* is the vapor pressure of the air at saturation (kPa); *e_a_* is the actual vapour pressure (kPa);
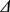
is the slope of the vapor pressure curve (kPa °C^−1^) and
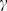
is the psychrometric constant (kPa °C^−1^). A complete set of equations is proposed by Allen et al. [13] to compute the parameters of Eq. (1) according to the available weather data and the time step computation, which constitute the so-called FAO-PM method. *G* can be ignored for daily time step computation. Using Eq. (1), we firstly calculated *ET_0_* for the 55 weather stations based on conventional meteorological observation data, and then estimated *ET_0_* for 138 counties through Kriging interpolation.

Actual evapotranspiration (ET) of croplands. Actual evapotranspiration of croplands was calculated by using the following equation,
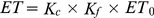
(2)where *ET* is actual evapotranspiration (mm); *K_c_* is crop coefficient; *K_f_* is soil moisture correction coefficient. The crop coefficient is largely dependent on crop varieties and planting patterns such as sowing density, fertilizer management, etc. So it varies largely in space and time and difficult to be collected, especially for the past, because information on grain varieties and growing observation data are not available. Alternatively, we assume that the temporal change of crop coefficient can be reflected by the grain yield coefficient (*GY_c_*) without distinguishing crop species in this study.

**Figure 2:**
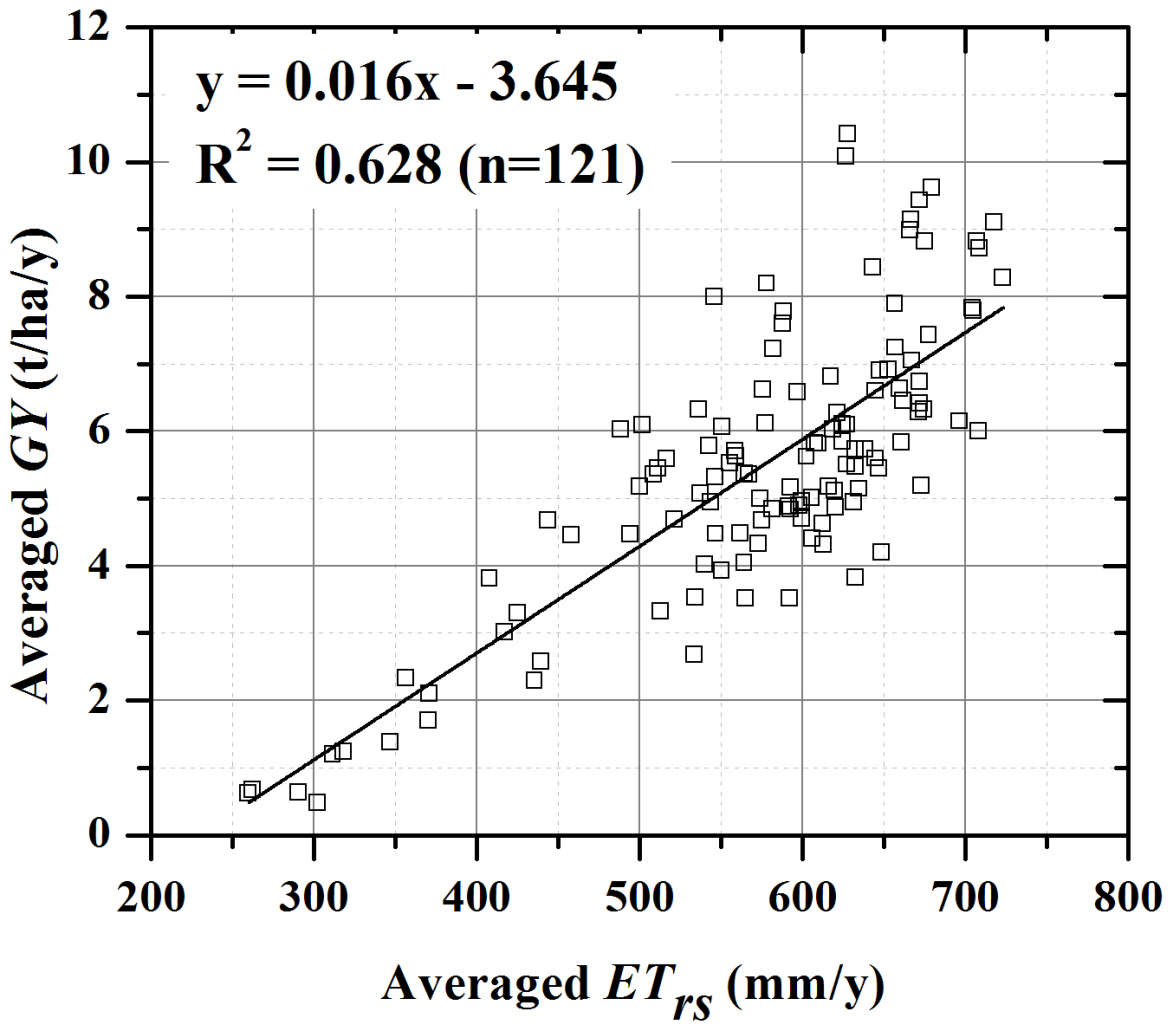
Relationship between remote sensing derived annual ET (*ET_rs_*) and grain yield (GY) from 2002 to 2008 of 121 major grain production counties.

The *GY_c_* is based on our analysis of the relationship between grain yield (GY) and water consumption, i.e. *ET*, in 121 counties of the all 138 counties by using the statistical yield for 2002–2008 and independent source of remote sensing derived *ET* data (thereafter, *ET*_rs_) for the same period. There are 17 counties, where the cultivated croplands mostly grow cotton and the grain croplands shares little to their total cultivated land, were removed from the correlation analysis of observed GY and *ET*_rs_ at county level. Figure 2 illustrated that the annual *ET* from the remote sensing *ET* products is significantly linearly correlated to the grain yield.

The grain yield coefficient *GY_c_* is calculated as follows,
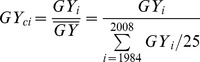
(3)where *GY_ci_* is grain yield coefficient of year *i*, *GY_i_* is grain yield of year *i* (t/ha), and 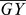
is the mean yield from 1984 to 2008 (t/ha). Therefore, Eq. (2) can be modified as,



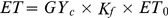
(4)Then, the soil moisture correction coefficient *K_f_* can be expressed as below,
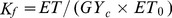
(5)


### Determination of *K_f_*

The annual *K_f_* of farmland was calculated using Eq. (5) on the basis of annual remote sensing *ET*_rs_ products (2002–2008) for each county, combined with annual *ET_0_* calculated for the same period. In this calculation, we used the areal weighted *ET_rs_* from grain crop lands for each county since the grain yield coefficient *GY_c_* only presents the water consumption and productivity from grain lands. Then we got the *K_f_* parameter for all the counties during the period 2002–2008.

There are no long-term soil moisture data available for the region, but it should reflect the annual precipitation and irrigation. So, we analyzed the correlations of annual *K_f_* with annual precipitation (*P*) and annual irrigation rate (*Irr*_rate_) of the 121 counties during the 7 years (totally, 847 samples), and resulted in an empirical equations below,
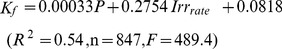
(6)where *P* is annual precipitation for each county (mm), *Irr*_rate_ is annual irrigation rate for each county which was defined as the effective irrigation area (*EIA*, km^2^) of a county to its total cultivated area (*A*, km^2^),



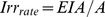
(7)With assumption of the regression coefficients in Eq. (6) keep stationary during the study period, we can get *K_f_* for each year in each county by using Eq. (6) from the mean annual precipitation and irrigation rates from 1984 through 2008.

### Agricultural Water Consumption and Irrigation Water Consumption

Agricultural water consumption (*WC_ag_*, km^3^) for each county was estimated as follows,
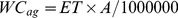
(8)


According to the water balance equation, total *ET* for the study period also can be estimated by the following formula,
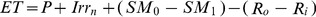
(9)where *Irr_n_* is effective irrigation water (mm); *SM_0_* is initial and SM*_1_* is final soil moisture (mm), when water balance for a relatively long period were calculated, 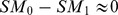; *R_o_* is outflow runoff (mm), *R_i_* is inflow runoff (mm) of croplands. In counties on the plain *R_i_* is basically equal to *R_o_*, and in mountainous counties, we estimate the difference between annual *R_o_* and *R_i_* using the method proposed by Ji et al. [14].



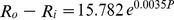
(10)According to the above analysis, the annual agricultural irrigation water of a county on plain area can be estimated as follows,
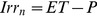
(11)while for the counties in mountainous region, it can be expressed as,



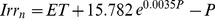
(12)The total irrigation water consumption *Irr_nc_* (km^3^), or net groundwater mining for each county can be estimated as follows,
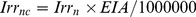
(13)


So, the annual decline rate of the groundwater table affected by agricultural irrigation can be simply estimated through,
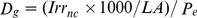
(14)where *D_g_* is the decline depth of groundwater (m); *LA* is land area (km^2^); *P_e_* is effective porosity, which ranges from 10 to 30% in the piedmont area, 5 to 20% in the middle alluvial plain, and 5 to 7% in the coastal plain, respectively [15]. In this study, uniform effective porosity of 25% was used across the plain area of the province.

### Validation and Sensitivity Analysis

The 5 years experimental data from Luancheng Agro-ecosystem Experimental Station as introduced earlier were employed to validate and evaluate the model’s performance. We assumed the five different irrigation treatments for the five years as different irrigation rate. Firstly, according to the different irrigation levels, such as rainfed, fully irrigation, 80% irrigation, 75% irrigation and 70% irrigation, we set the irrigation rates (*Irr*_rate_) of the 5 treatments as 0, 1.0, 0.8, 0.75 and 0.7, respectively. Then, the key parameters of *GY_c_* and *K_f_* were calculated according to Eq. (3) and (6). *GY_c_* ranged from 0.44 to 1.30 and *K_f_* from 0.20 to 0.55, respectively. Finally, we applied all the yield data, *P,* and *ET_0_* to the model and calculated the *ET* for different treatments in each year.

**Figure 3:**
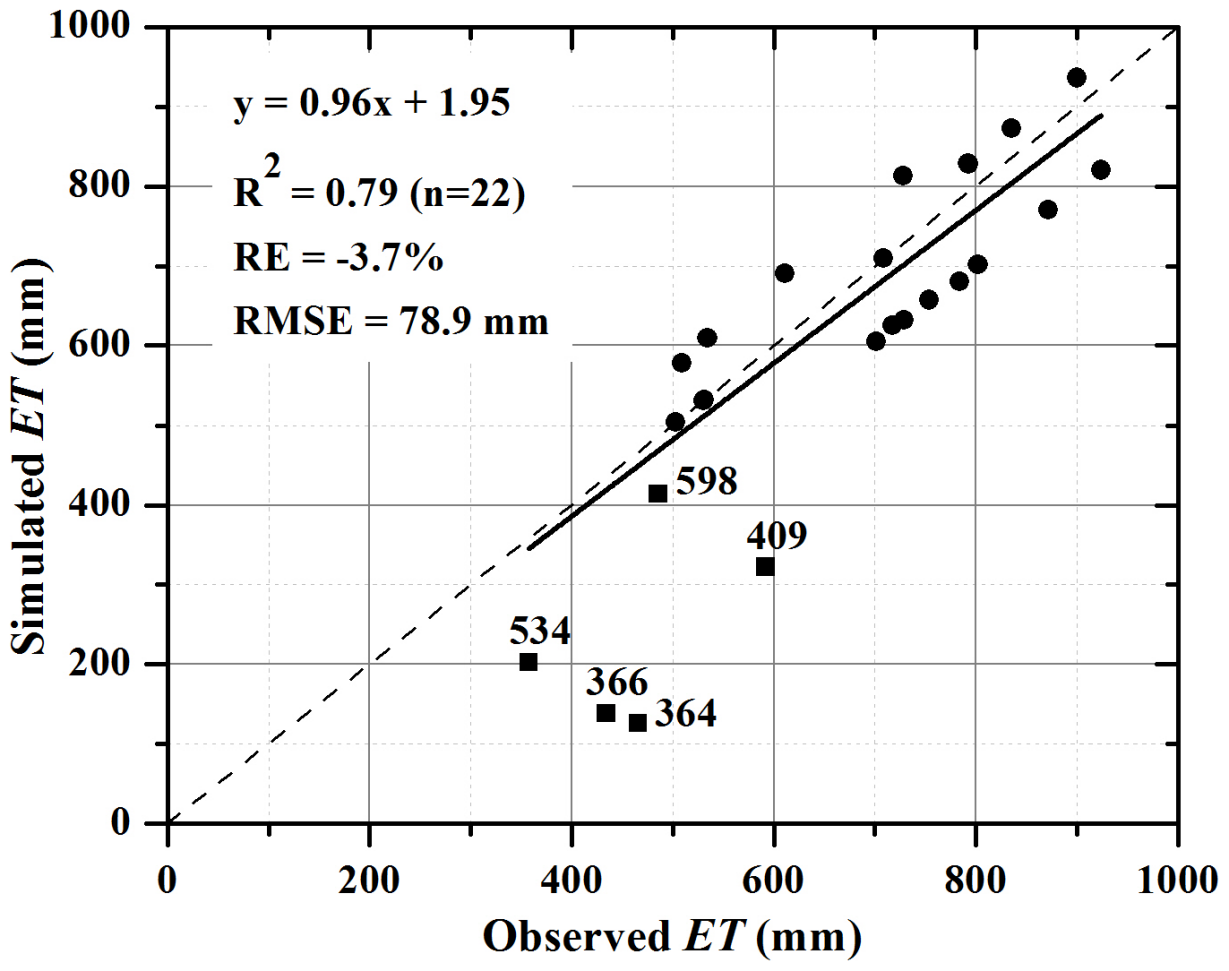
Validation of the model by using field experimental data from Luancheng Agro-ecosystem Experimental Station, Chinese Academy of Sciences. The numbers associated with the 5 points, representing rain-fed treatments, at the lower part refer to annual precipitation. The data for 3 filled blocks (409 mm, 366 mm, and 364 mm) are not included in the regression line because of the large deviations to the observed ET, indicating the model will underestimate actual ET for the non-irrigated lands when annual P less than around 400 mm.

The comparison of calculated *ET* with field observed *ET* through soil water balance demonstrated a quite good consistency (Figure 3) except for 3 rainfed treatments in dry years. The relative bias for the 22 samples is only −3.7% and RMSE is 78.9 mm. But for the rainfed treatment in dry years, without supplementary irrigation the grain yield will be largely dependent the occurrence of rainfall on by both amount and timing, which induces uncertainty of the grain yield response to rainfall. In our studies, the main purpose is to give a good projection of the groundwater depletion because of irrigation pumping in past decades. We judge that the bias happened in rainfed cropland will have minor effects on this objective.

**Figure 4:**
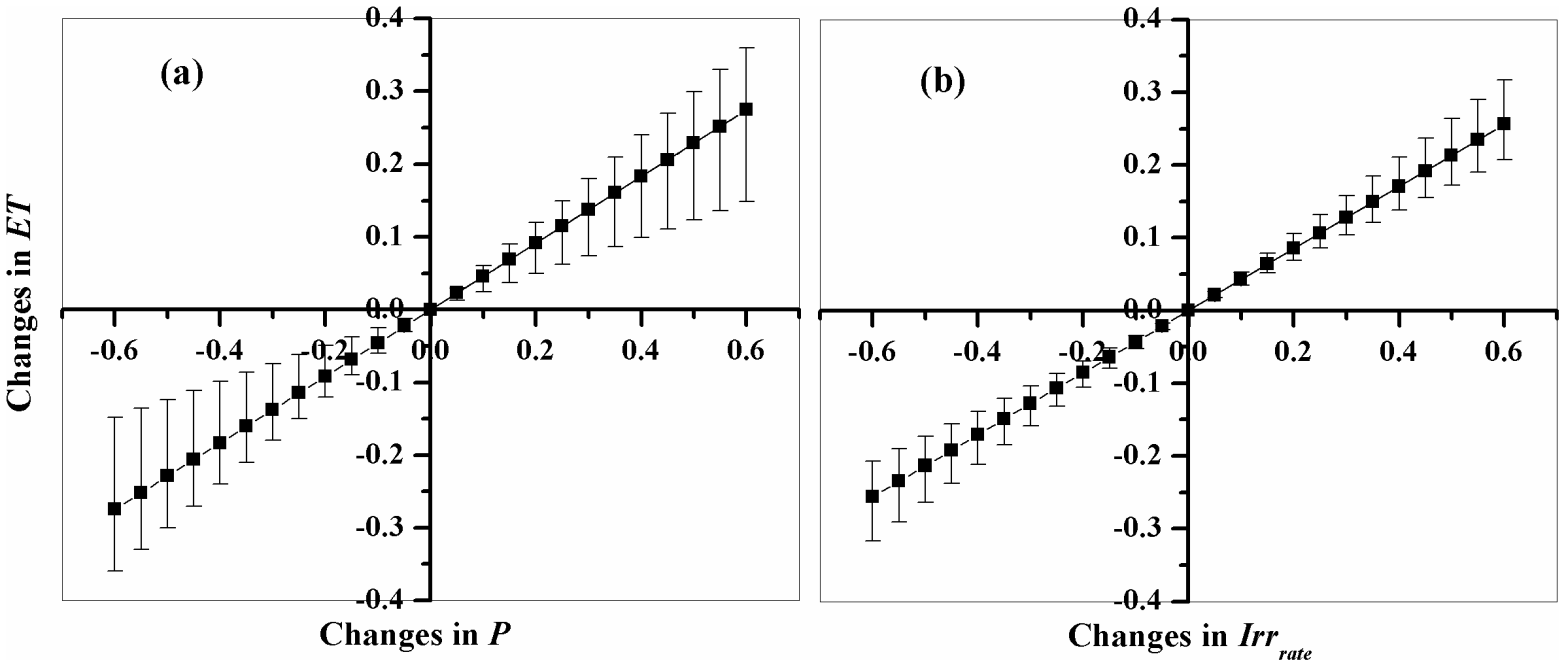
Sensitivities of estimated ET to the changes in annual precipitation (a), and to the changes in irrigation rates (b). The error bars indicates the range of different irrigation rates in (a), and range of different annual precipitation in (b), respectively.

In order to evaluate the effectiveness of parameter *K_f_*, we conducted a sensitivity analysis of the estimated ET to the key variables in Eq. 6, i.e. precipitation (*P*) and irrigation rate (*Irr*_rate_). Figure 4 illustrated the responses of ET change to changes in *P* under different irrigation rates (Figure 4a) and to changes in irrigation rate under different annual precipitations (Figure 4b). *Δ*ET*/ΔP* varies from 0.32–0.67 when irrigation rate varies from 100% to zero (Figure 4a); the dependence of ET on *P* decreases as irrigation rate increases. While, the dependence of ET on irrigation rate shows a smaller range, *Δ*ET*/ΔIrr*_rate_ varies from 0.35–0.53 when annual precipitation decreases from 700 mm to 200 mm. So, the soil wetness parameter *K_f_* is sensitive enough to annual *P* and irrigation rate, and the model can reflect good responses of estimated ET to precipitation and irrigation at an annual base.

## Results

### *ET_0_*, *ET* and AWC

**Figure 5:**
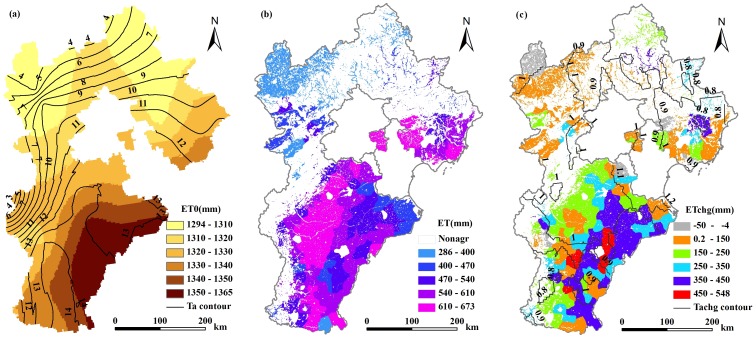
Distribution of annual mean *ET*_0_ (a), actual ET (b), and changes in averaged ET for the period of 1984–1993 to 1999–2008 (c). The contour lines in (a) indicate the distribution of annual mean temperature (Ta); contour lines in (c) indicate the change of annual air temperature (Tachg) for the same time slices.

Mean annual ET_0_ (1984–2008) ranged from 1,294 mm to 1,365 mm, decreasing gradually from southeast to northwest and showing a similar spatial pattern to air temperature (Figure 5a). And mean annual *ET* of croplands in each county ranged from 286 to 674 mm (Figure 5b). *ET* has significantly increased during the past 25 years except for a few counties located in mountainous regions. Increasing *ET* from croplands is attributed mainly to intensified agricultural activity, such as changes in sowing density, irrigation rate, etc., especially in the plain areas, and partly to increasing temperature (Figure 5c). Decreased *ET* was detected in some mountainous counties as was shown in Figure 5c, this phenomenon may reflect the effects of the state policy so-called ‘Grain to Green’, which was launched in the end of 1990s to prevent the land desertification and sand storm through returning cropland to forest or grassland. Mean annual agricultural water consumption (AWC) for the counties ranged from 50 million m^3^ to 550 million m^3^ in Hebei province during the period from 1984 to 2008. The total water consumption for agricultural grain production was estimated as much as 864 km^3^ in Hebei province during the 25 years.

### Net consumption of Irrigation Water

**Figure 6:**
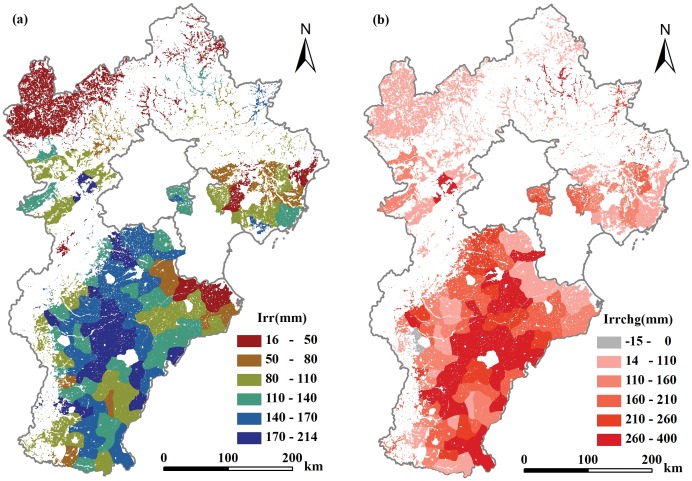
Annual mean net groundwater irrigation in 1984–2008 (a), and its change (b) from the periods of 1984–1993 to 1999–2008.

Mean annual net irrigation water (mainly groundwater, *Irr_n_*) for each county ranged from 16 to 214 mm (Figure 6a) during the study period, in other words, the groundwater table changes would response to these numbers. The counties at the southeast part of the low plain region showed large increase in irrigation water consumption during the period of 1999–2008 compared with that in 1984–1993 (Figure 6b). That region used to be saline soil and shallow groundwater. The grain productivity has increase greatly during past 3 decades due to the efforts in drainage system construction and irrigation technology evolution. The total net groundwater consumption for irrigation (*Irr_nc_*), calculated through Eq. (11 & 12) for the plain area during the study period was projected as 113 km^3^ with the mean annual value for each county ranging from 2.7 million m^3^ to 140 million m^3^.

## Discussion and Conclusions

**Figure 7:**
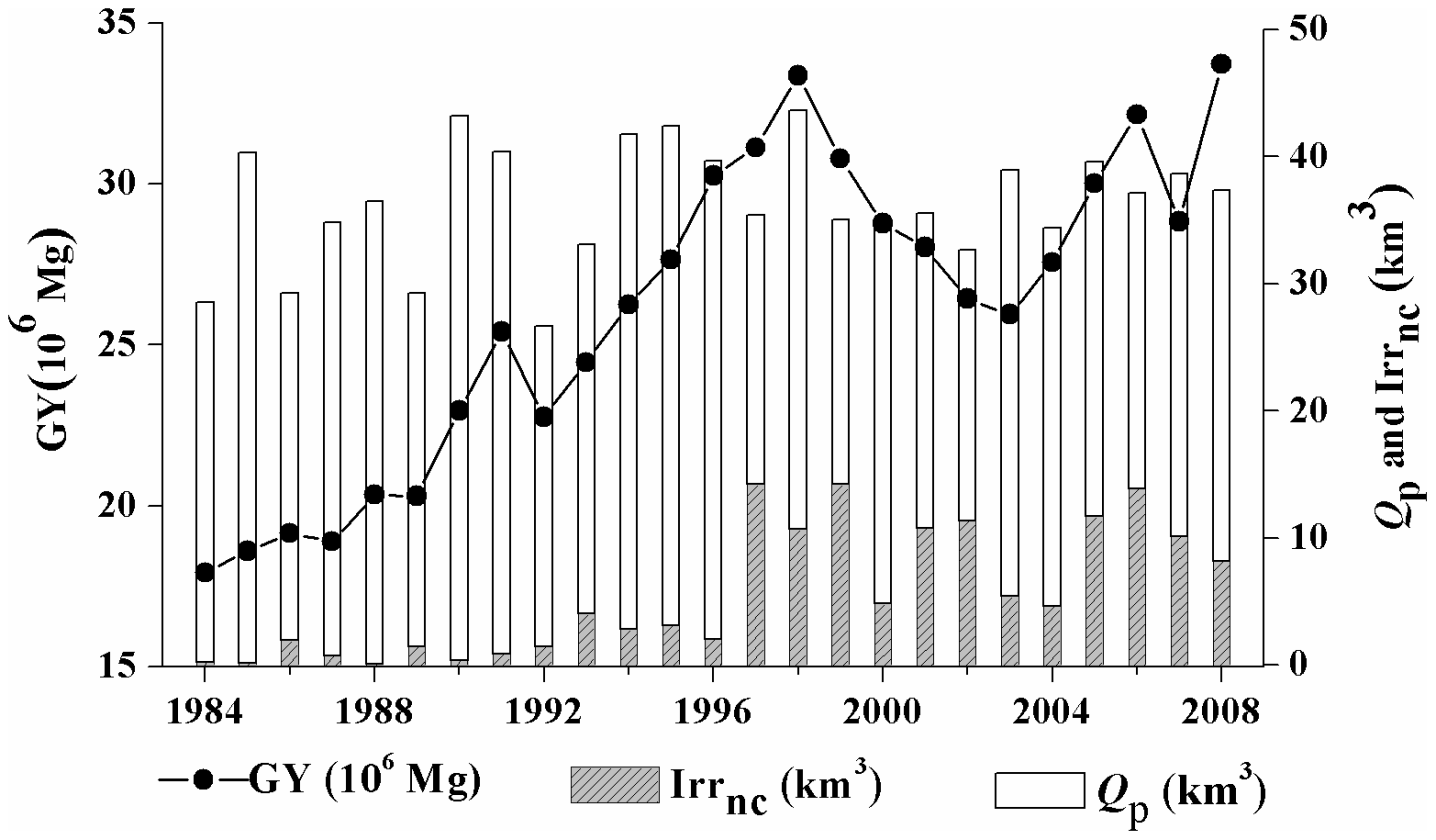
Inter-annual variations of precipitation (*Q_p_*), net groundwater consumption (*Irr_nc_*), and grain yield (*GY*) in Hebei Province (1984–2008).

Water shortage has become a major limiting factor for the sustainable development of agriculture in Hebei. The estimation of agricultural water and groundwater irrigation net consumption will provide scientific information for developing efficient irrigation practices to improve crop water productivity. During the study period from 1984 to 2008, the 138 counties in Hebei province produced a total of 6.1×10^8 ^Mg of grain, and consumed 864 km^3^ of water (with an average of 34.6 km^3^/y), including 139 km^3^ of groundwater. The AWC estimating result was close to the fresh water footprint of agriculture, which was calculated by using of Gini coefficient and Theil index accounting for 33.4 km^3^ in Hebei province in 2007 [16]. Figure 7 shows the variations of annual grain yield (*GY*), water from precipitation (*Q_p_*) and groundwater irrigation net consumption (*Irr_nc_*) in Hebei from 1984 to 2008. In terms of spatial distribution, the grain yield and AWC in the southeastern part of the province are significantly higher than those in the northwest.

Based on the linear correlation of *ET* and grain yield of each county (Figure 2), we estimated the grain yield without groundwater consumption, and subtracted this rain water fed yield from the actual statistical yield to obtain the grain yield gain (GYG) benefited from groundwater irrigation. It is found that the accumulated grain yield gain in the 25 years would be 1.9 × 10^8^ Mg, which accounts for 31% of the province’s total grain production during the same period.

**Figure 8:**
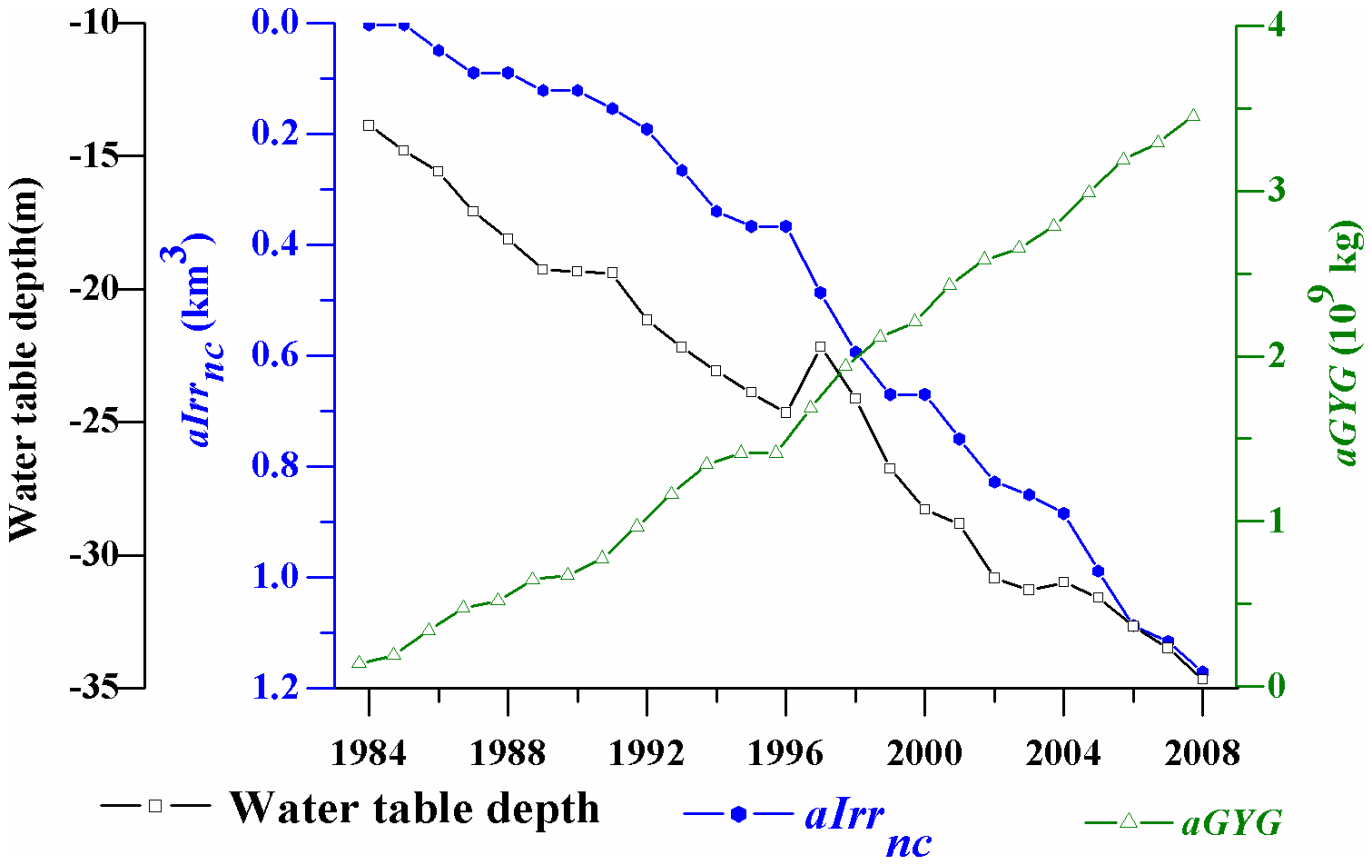
Accumulated net groundwater consumption (a*Irr_nc_*), grain yield gain (a*GYG*), and observed groundwater table depth change for Luancheng county (1984–2008).

In addition, we took Luancheng County (location shown in Figure 1) as a typical example to analyze the trade-off between groundwater consumption and grain yield gain. The irrigation rate of Luancheng County has reached to more than 90% since the beginning of 1980s. Although exploitation of groundwater ensured a stable increase in grain production, the groundwater table in Luancheng fell 20.82 m from 1984 to 2008 due to continual over-pumping (Figure 8). The total groundwater consumption in Luancheng County estimated by the model accounted for 1.2 km^3^ in the 25 years, which could cause the underground water table falling of 13.5 m in Luancheng area during the same period. Our estimation attributes the agricultural irrigation for grain production contributed 65% of the groundwater depletion in this county.

Large-scale mining of groundwater in Hebei Province began in the 1970s, the rapid socio-economic development consumed a large amount of groundwater in recent decades, the consumption of agricultural irrigation accounted for 77% of the total. Due to over-exploitation of groundwater, the underground water level was steadily declining. The total groundwater consumption in the plain area during the study period was estimated as 113 km^3^, which could cause an average groundwater falling of 7.4 m over the plain. This estimation is greatly agree with the results reported by Cao [17], who used a numerical groundwater flow model to simulate the groundwater pumping and water table decline over the Hebei plain.

In this study, we aimed to estimate the AWC and groundwater irrigation consumption of Hebei province in recent decades using a simple model. The model proposed in this study need only the basic meteorological data and annual grain yield data. Based on some important assumptions the model can give good estimates of agricultural water consumption and net groundwater consumption for grain food production, and meet the study objectives well. However, we would like to call the audience attention to the uncertainties included in this study. First of all, we used a grain yield coefficient to substitute the crop coefficient in calculation of actual *ET*. This assumption ignored the differences in crop varieties, planting and field managements, irrigation methods, etc. and might cause some deviations of the results. Second, soil moisture of each year is different, but for any region it remains basically unchanged over the long term. The *ET* calculated by Eq. (1) for each year therefore varies, but it is reasonable to use Eq. (1) to calculate the sum and mean annual *ET* over the 25 years period. The *ET* products derived from remote sensing data and economic statistics data also contain some uncertainties [18], these sources of uncertainty may affect the accuracy of this study.

However, through comparing our estimations with the observed *ET* and groundwater depth at Luancheng county and further with the independent simulation over Hebei Plain [17] we have great confidence to believe the method proposed in this study could be extrapolated and applied to other regions where limited data such as meteorological and yield census data are available. Also it may be used in those regions for assessing the water footprint or aiding better water management for sustainable development.
